# Supplementary material for: Spatio-temporal distribution and socioeconomic inequality of low birthweight rate in China from 1992 to 2021 and its predictions to 2030
Source: PLoS One. 2025 Jan 7;20(1):e0310944. doi: 10.1371/journal.pone.0310944 (PMC11706412; doi:10.1371/journal.pone.0310944)
Supplement: S1 Table — APC, annual percentage change; AAPC, average annual percentage change; CI, confidence intervals; *p < 0.05, **p < 0.01. (DOC) [file pone.0310944.s001.doc]

**Supplementary table 1 Joinpoint regression of low birthweight rate in 30 provinces of China from 2002 to 2021.**

| Province | Year | APC (95%CI) | AAPC (95% CI) | Province | Year | APC (95%CI) | AAPC (95% CI) |
| --- | --- | --- | --- | --- | --- | --- | --- |
| Xinjiang | 2002-2009 | 12.44 (9.95, 14.99)** | 8.22 (6.58, 9.89)** | Guizhou | 2002-2004 | -27.09 (-41.17, -9.65)** | 3.01 (0.00, 6.11)* |
|  | 2009-2015 | -0.33 (-4.00, 3.48) |  |  | 2004-2012 | 0.38 (-2.46, 3.30) |  |
|  | 2015-2021 | 12.39 (9.24, 15.62)** |  |  | 2012-2018 | 16.93 (11.45, 22.67)** |  |
| Tianjin | 2002-2006 | -0.81 (-7.41, 6.26) | 6.62 (3.02, 10.35)** |  | 2018-2021 | 7.83 (-3.14, 20.04) |  |
|  | 2006-2009 | 37.87 (10.89, 71.40)** |  | Guangdong | 2002-2021 | 3.00 (2.63, 3.37)** | 3.00 (2.63, 3.37)** |
|  | 2009-2021 | 2.43 (1.12, 3.75)** |  | Fujian | 2002-2004 | 7.52 (-1.67, 17.58) | 2.99 (1.94, 4.04)** |
| Neimenggu | 2002-2021 | 5.93 (5.26, 6.61)** | 5.93 (5.26, 6.61)** |  | 2004-2012 | 0.12 (-1.06, 1.33) |  |
| Hunan | 2002-2010 | 5.89 (4.66, 7.14)** | 5.70 (4.66, 6.76)** |  | 2012-2021 | 4.60 (3.75, 5.45)** |  |
|  | 2010-2016 | 3.04 (0.60, 5.54)* |  | Guangxi | 2002-2021 | 2.56 (2.13, 3.00)** | 2.56 (2.13, 3.00)** |
|  | 2016-2021 | 8.68 (6.10, 11.32)** |  | Anhui | 2002-2005 | -11.52 (-19.84, -2.33)* | 2.35 (0.31, 4.44)* |
| Hubei | 2002-2004 | -8.33 (-23.58, 9.95) | 4.60 (2.70, 6.54)** |  | 2005-2011 | -0.71 (-5.00, 3.78) |  |
|  | 2004-2021 | 6.24 (5.57, 6.92)** |  |  | 2011-2021 | 8.88 (7.22, 10.57)** |  |
| Jiangsu | 2002-2013 | 3.05 (1.69, 4.43)** | 4.40 (3.27, 5.55)** | Shanxi | 2002-2009 | -3.19 (-4.61, -1.74)** | 2.09 (0.82, 3.38)** |
|  | 2013-2021 | 6.28 (4.02, 8.59)** |  |  | 2009-2019 | 3.36 (2.32, 4.40)** |  |
| Yunnan | 2002-2009 | 7.00 (5.00, 9.04)** | 4.09 (3.26, 4.93)** |  | 2019-2021 | 15.58 (3.48, 29.10)* |  |
|  | 2009-2021 | 2.43 (1.58, 3.29)** |  | Jilin | 2002-2006 | -20.84 (-26.54, -14.71)** | 1.92 (0.29, 3.58)* |
| Ningxia | 2002-2021 | 3.99 (3.11, 4.87)** | 3.99 (3.11, 4.87)** |  | 2006-2021 | 9.03 (7.95, 10.12)** |  |
| Beijing | 2002-2005 | 0.87 (-3.99,5.97) | 3.92 (3.12, 4.73)** | Jiangxi | 2002-2006 | -1.04 (-4.53, 2.58) | 1.65 (-0.24, 3.58) |
|  | 2005-2021 | 4.50 (4.11, 4.90)** |  |  | 2006-2009 | 9.84 (-1.95, 23.03) |  |
| Heilongjiang | 2002-2005 | -1.29 (-5.85, 3.48) | 3.86 (2.22, 5.53)** |  | 2009-2016 | -2.73 (-4.58, -0.85)* |  |
|  | 2005-2008 | 30.22 (18.48, 43.11)** |  |  | 2016-2021 | 5.45 (2.81, 8.16)** |  |
|  | 2008-2016 | -2,88 (-4.09, -1.64)** |  | Shandong | 2002-2013 | -2.59 (-3.56, -1.60)** | 1.12 (0.28, 1.96)** |
|  | 2016-2021 | 4.09 (1.92, 6.31)** |  |  | 2013-2021 | 6.44 (4.72, 8.19)** |  |
| Shanghai | 2002-2021 | 3.68 (3.26, 4.11)** | 3.68 (3.26, 4.11)** | Tibet | 2002-2021 | 0.15 (-3.03, 3.44) | 0.15 (-3.03, 3.44) |
| Sichuan | 2002-2011 | -0.95 (-2.78, 0.92) | 3.48 (2.32, 4.65)** | Liaoning | 2002-2010 | -4.66 (-6.29, -3.00)** | -0.44 (-1.31, 0.44) |
|  | 2011-2021 | 7.63 (5.93, 9.36)** |  |  | 2010-2021 | 2.75 (1.66, 3.86)** |  |
| Hainan | 2002-2006 | -4.64 (-9.61, 0.60) | 3.44 (1.40, 5.52)** | Henan | 2002-2011 | -6.87 (-8.00, -5.73)** | -0.57 (-1.29, 0.16) |
|  | 2006-2019 | 3.98 (2.95, 5.03)** |  |  | 2011-2021 | 5.47 (4.38, 6.57)** |  |
|  | 2019-2021 | 17.65 (-0.66, 39.35) |  | Qinghai | 2002-2009 | -6.94 (-10.88, -2.82)** | -1.35 (-3.15, 0.48) |
| Chongqing | 2002-2013 | -1.36 (-4.07, 1.44) | 3.23 (0.88, 5.63)** |  | 2009-2021 | 2.06 (0.13, 4.03)* |  |
|  | 2013-2021 | 9.88 (5.02, 14.96)** |  | Hebei | 2002-2014 | -3.11 (-3.65, -2.56)** | -2.20 (-2.73, -1.68)** |
| Zhejiang | 2002-2004 | -8.36 (-17.75, 2.10) | 3.18 (2.06, 4.31)** |  | 2014-2021 | -0.64 (-1.89, 0.62) |  |
|  | 2004-2021 | 4.63 (4.23, 5.03)** |  | Gansu | 2002-2012 | -8.81 (-9.95, -7.65)** | -2.61 (-3.47, -1.74)** |
| Shaanxi | 2002-2013 | -0.44 (-2.09, 1.24) | 3.04 (1.63, 4.47)** |  | 2012-2021 | 4.77 (3.23, 6.33)** |  |
|  | 2013-2021 | 8.03 (5.14, 11.00)** |  |  |  |  |  |

APC, annual percentage change; AAPC, average annual percentage change; CI, confidence intervals; **p* < 0.05, ***p* < 0.01.
