# Supplementary material for: Spatio-temporal distribution and socioeconomic inequality of low birthweight rate in China from 1992 to 2021 and its predictions to 2030
Source: PLoS One. 2025 Jan 7;20(1):e0310944. doi: 10.1371/journal.pone.0310944 (PMC11706412; doi:10.1371/journal.pone.0310944)
Supplement: S2 Table — ARIMA, autoregressive integrated moving average; BIC, bayesian information criterion. (DOC) [file pone.0310944.s002.doc]

Supplementary Table 2 The results of the ARIMA models.

| Area | ARIMA | Ljung-Box Q test | | BIC |
| --- | --- | --- | --- | --- |
|  |  | χ^2^ value | *P* value |  |
| Total | (0,2,1) | 0.3298 | 0.5658 | -14.25 |
| Eastern regions | (0,2,2) | 0.2357 | 0.6273 | -20.78 |
| Central regions | (0,2,1) | 0.5106 | 0.4749 | -20.42 |
| Western regions | (0,2,1) | 0.2538 | 0.6144 | -14.99 |

ARIMA, autoregressive integrated moving average; BIC, bayesian information criterion.
