# Supplementary material for: Spatio-temporal distribution and socioeconomic inequality of low birthweight rate in China from 1992 to 2021 and its predictions to 2030
Source: PLoS One. 2025 Jan 7;20(1):e0310944. doi: 10.1371/journal.pone.0310944 (PMC11706412; doi:10.1371/journal.pone.0310944)
Supplement: Attachment — Submitted filename: Reviewer.pdf [file pone.0310944.s004.pdf]

Reviewer's report

Thank you for inviting me to review this important manuscript. **Accept this paper with minor adjustment**

Specific comments

Kindly include the keywords of this study

Discussion

Kindly explain the reason for the increment in LBWR in China.

Elaborate more and compare the findings with other Asian countries

Line 239: "…..LBWR has increased in the last 10 years". Kindly include the reference.

Don't just discuss the limitations of the study, also add few points regarding the strength

Highlight and discuss what the government has done so far regarding the steady rising of LBWR

Finally, add some recommendations to your conclusion
