# Supplementary material for: Spatio-temporal distribution and socioeconomic inequality of low birthweight rate in China from 1992 to 2021 and its predictions to 2030
Source: PLoS One. 2025 Jan 7;20(1):e0310944. doi: 10.1371/journal.pone.0310944 (PMC11706412; doi:10.1371/journal.pone.0310944)
Supplement: Attachment — Submitted filename: Response to Reviewers.doc [file pone.0310944.s005.doc]

**Response to Reviewers**

**PONE-D-24-28886**

**Spatial-temporal distribution and socioeconomic inequality of low birthweight rate in China from 1992 to 2021 and its predictions to 2030**

**Reviewer 1**

1.The manuscript is technically sound, and the data do support the conclusions.

2.The statistical analysis has been performed appropriately and rigorously.

3.The authors have made all data underlying the findings in their manuscript fully available.

4. The manuscript is presented in an intelligible fashion and written in standard English.

Re: Thank you for the corrections to the English phrases in the article. Keywords have been added and references have been checked. See Tracking Changes manuscript for other revisions.

**Reviewer 2**

Kindly include the keywords of this study

Re: Add keywords.

Discussion

Kindly explain the reason for the increment in LBWR in China.

Re: The reasons for the increase in LBWR are discussed considerably in paragraphs 3-4 of the Discussion section, with possible factors being the increase in ozone concentration, the increase in the prevalence of overweight and obesity among pregnancy-age women, the delay in the reproductive age of females, the increase in prenatal complications, the increase in the proportion of alcohol consumption among pregnant women, etc. **Pages 7-8, lines 267-304.**

Elaborate more and compare the findings with other Asian countries

Re: Added reference 31, which is the most recently published evidence of global trends in LBWR, including most countries worldwide (also including Asia). Unfortunately, except for the data from reference 31 and Korea and Japan provided in the original article, there is little evidence of recent trends from Asia. **Page 7, lines 247-251.**

Line 239: “…..LBWR has increased in the last 10 years”. Kindly include the reference.

Re: Added references “[19,22-23,30-31]”. **Page 7, line 263.**

Don’t just discuss the limitations of the study, also add few points regarding the strength

Re: The strengths of this study were added at the end of the discussion. **Pages 10-11, lines 397-410.**

Highlight and discuss what the government has done so far regarding the steady rising of LBWR

Re: A related discussion has been added to the penultimate paragraph of the Discussion section. **Page 10, lines 374-396.**

Finally, add some recommendations to your conclusion

Re: Some suggestions have been added to the conclusion section. **Page 11, lines 433-441.**
